# Influencing factors comparing different vault groups after phakic implantable collamer lens implantation: review and meta-analysis

**DOI:** 10.1186/s12886-024-03325-9

**Published:** 2024-02-15

**Authors:** Pengcheng Zhang, Chenjun Guo, Song Wang, Wenshan Jiang, Dan Wang, Hong Yan

**Affiliations:** 1 Shaanxi Eye Hospital, Xi’an People’s Hospital (Xi’an Fourth Hospital), Affiliated People’s Hospital of Northwest University, 710004 Xi’an, China; 2 Department of Ophthalmology, General Hospital of Central Theater Command, PLA, 430070 Wuhan, China; 3 Department of Ophthalmology, Tangdu Hospital, Air Force Medical University, 710032 Xi’an, China; 4 Department of Ophthalmic Center, General Hospital of Xinjiang Military Region, 830099 Urumqi, China

**Keywords:** Implantable intraocular lens, Vault, Influencing factors, Systematic review, Meta-analysis

## Abstract

**Background:**

Studies on the factors affecting vault after posterior chamber phakic Implantable Collamer Lens (ICL) have been carried out, but most of them are single-centered and subjective selections of parameters. The present study aimed to systematically analyze the factors for vault.

**Methods:**

A systematic review of case series, case-control, and cohort studies derived from the articles published in PubMed, the Cochrane Library, Embase, Web of Science, CNKI, CBM, Wanfang and VIP, as well as ClinicalTrials, which were conducted to search for studies on factors of vault using four core terms: phakic intraocular lenses, vault, risk factor and observational study, from January 01, 1997, to February 20, 2023. The included studies were meta-analyzed quantitatively and described qualitatively. Subsequently, meta-regression and subgroup analysis were used.

**Results:**

We identified 13 studies (1,607 subjects), and 14 factors were considered. Meta-analysis showed that anterior chamber depth (ACD), horizontal corneal white-to-white (hWTW), ICL-size, and age are dual effects of the abnormal vaults; anterior chamber volume (ACV) and lens thickness (LT) are a one-way effect; while axial length (AL), ICL- spherical equivalent (ICL-SE) and Km are insignificant. In addition, descriptive analysis of anterior chamber angle (ACA), horizontal sulcus to sulcus (hSTS), ciliary processes height (T value), crystalline lens rise (CLR), and gender showed that all factors except gender tend to have significant effects on vault. Sensitivity analysis showed stable combined results. Country and design respectively affect the heterogeneity in ACD and ICL-size at low vault, while design affects the heterogeneity in ACD at high vault. No publication bias exists.

**Conclusions:**

Vault after ICL is related to multiple factors, especially anterior segmental biologic parameters, and they are weighted differently. We hope to provide a reference for the selection and adjustment of ICL.

## Background

Modern refractive surgery mainly includes keratomileusis and intraocular refractive surgery [1]. Due to its good correction of keratoconus or hyperopia, the phakic intraocular lens, particularly the Vision Implantable Collamer Lens (ICL) V4/V4c, has received much attention [2, 3]. ICL implantation is an intraocular procedure which provides superior visual quality, minimal complications and is reversible [4]. Its safety, efficacy and long-term stability have been universally proven [5, 6].

However, long-term follow-up is required after ICL implantation, with a focus on site identification to assess safety. Vault, as an important parameter of ICL position in the posterior chamber and assessment of safety [7], refers to the maximum vertical distance between the apex of the anterior surface of the crystalline lens and posterior surface of ICL [8]. The ideal vault is 250–750 μm, which means an abnormal vault if beyond this range [9]. Too low a vault can easily cause cataracts, too high interferes with the anterior chamber, rubs the iris, and induces persistent high intraocular pressure, uveitis, etc. [10, 11].

Currently, studies on the factors affecting vault have been carried out, but most of them are single-centered and subjective selections of parameters, lacking a more comprehensive and systematic study, which is the vital feature of this review. Except for anterior chamber depth (ACD) and horizontal corneal white-to-white (hWTW) [12, 13], all other factors have not been elucidated and the weights are not yet known. Aiming to provide clues for preoperative ICL selection and vault prediction, this study undertook a systematic evaluation and meta-analysis, focusing on two questions: (1) What are the factors influencing high or low vault compared with the normal one, and what are the similarities and differences? (2) How can the controllable part of the above factors be avoided to improve the accuracy of ICL selection?

## Methods

### Search strategy

According to prespecified criteria [14] outlined by the preferred reporting items for systematic reviews and meta-analyses (PRISMA) guidelines, this study protocol was registered with PROSPERO (No. CRD42023403759). Two investigators (PZ and CG) independently searched eight databases to identify all the eligible literature from January 01, 1997, to February 20, 2023: PubMed, the Cochrane Library, Embase, Web of Science, Chinese National Knowledge Infrastructure (CNKI), Chinese Biomedical Literature (CBM), Wanfang and VIP, by a combination of Medical Subject Headings [MeSH] terms and keywords. The ClinicalTrails and China Clinical Trials Registry were also searched manually for unpublished relevant literature. It contained four core components, linked using the AND operator: (1) phakic intraocular lenses (e.g. phakic, implantable collamer lens, ICL, intraocular lens implantation); (2) vault (e.g., arch height, arch highness, high arch); (3) risk factor (e.g., influenc* factor, relate*, predict*); (4) observational study (e.g., case-control study, cohort study, case series study). Search terms were reviewed by an independent specialist (HY) to ensure its comprehensive and relevant.

### Search selection and data extraction

Two researchers (PZ, CG) independently performed literature screening and data extraction, and appraisal of study quality using the same criteria. Duplicates were eliminated in Endnote (version X9), then titles and abstracts were screened for eligibility, a full text was read for re-screening, and studies were finally included in the quantitative analysis. Disagreements were adjudicated by consultation between the two reviewers and arbitration by a third reviewer (HY). Inclusion criteria: (1) design types were observational studies, including case series, case-control or cohort studies; (2) the study subjects had refractive error and received ICL implantation, regardless of gender or age; (3) were divided into groups based on vault, which of 250–750 μm were considered as normal vault group, larger or less than this range as high and low vault, respectively; the difference in age between groups was less than 5 years, and they were admitted to the same hospital during the same period; (4) had full text available. Exclusion criteria: (1) reviews, meta-analyses, case reports, letters, conference proceedings; (2) with inconsistent study purposes or designs; (3) publications with low quality or duplicated, no control group, or incomplete data; (4) studies with grouping basis or definition of factors significantly different from the general criteria or most study criteria.

### Quality assessment

The quality of the included literature was evaluated using the Newcastle-Ottawa Scale (NOS) [15]. The NOS consists of 8 entries in 3 dimensions with a total score of 9. Higher scores are associated with better quality, with a score greater or equal to six being of higher quality [16].

### Statistical analysis

The combined effect sizes for continuous variables were expressed utilizing standardized mean differences (SMD) with a 95% confidence interval (CI). Heterogeneity between studies was tested using Q statistic and I [2] test. When the I [2] value was more than 50%, which indicated a significant heterogeneity, the random-effects model was used. Otherwise, the fixed-effects model was chosen. The stability of the results was tested by sensitivity analysis. The heterogeneity was considered large when I [2] was larger than 75%, and meta-regression and subgroup analysis were performed to assess significant factors of it. Statistical analyses were performed with the software Review Manager 5.4 (Cochrane Collaboration, London, UK) and STATA/SE version 16 (Stata Corporation, College Station, TX, USA). The test level was α = 0.05.

## Results

**Fig. 1:**
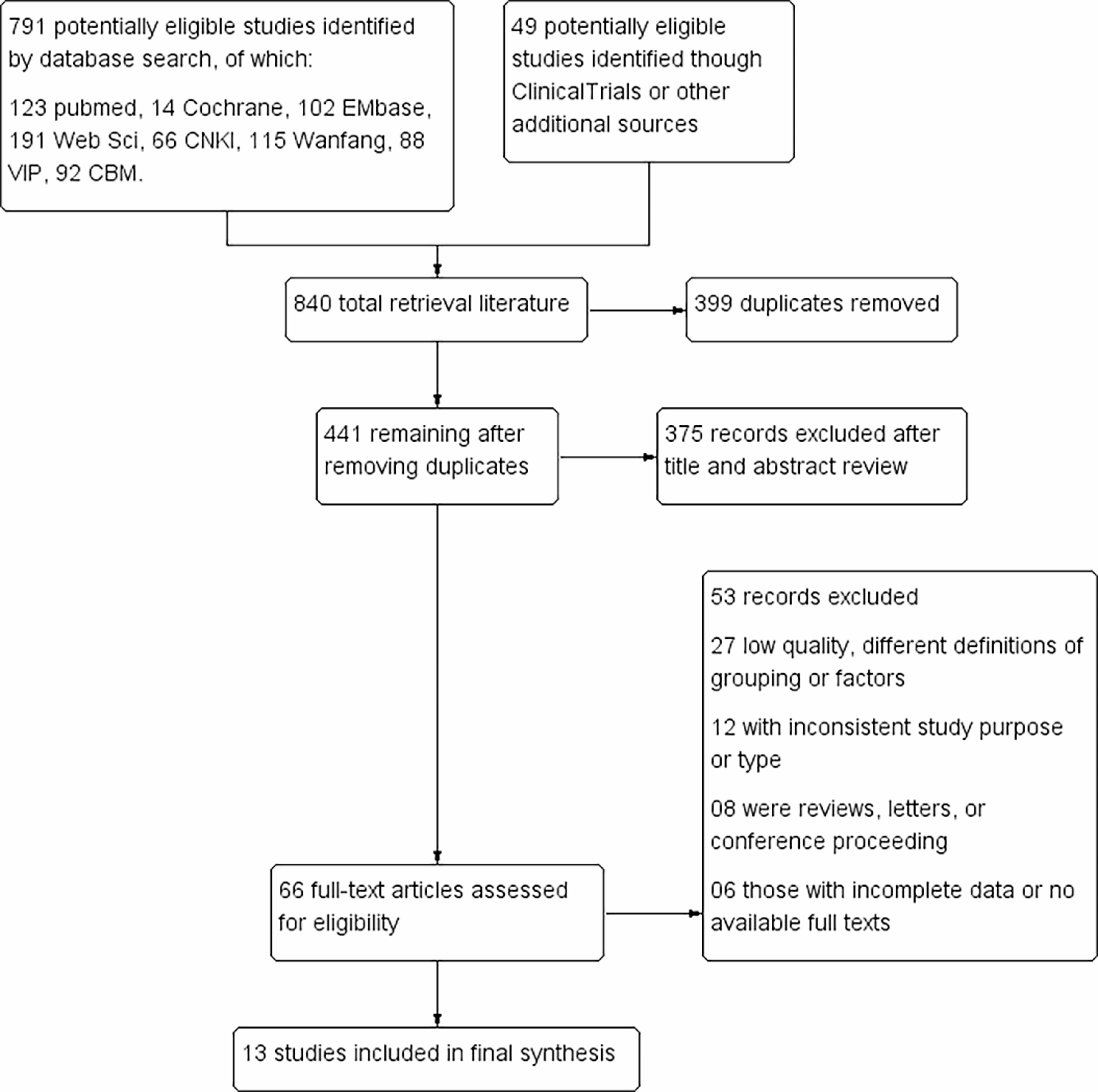
PRISMA flow diagram

A total of 840 articles were identified by computer searching, and 13 studies [9, 17–28] finally met the inclusion criteria after screening and extraction (Fig. 1), all of which were single-center studies without the combination of other ocular diseases affecting visual acuity.

### Study characteristics and quality assessment

Table 1 summarizes the characteristics of the studies included in the evidence synthesis. Thirteen included studies were all observational (8 case series [9, 20, 22–27], 4 case-control [18, 19, 21, 28], and 1 cohort [17]); Five of them were in English and 8 in Chinese; For measurement of vault, 6 studies used Pentacam [18, 21–25] and 7 ones used AS-OCT [9, 17, 19, 20, 26–28], while ultrasound biomicroscope (UBM) was also used as a complementary measure of correlative indicators, such as horizontal sulcus to sulcus (hSTS), lens curvature and ciliary processes height (T-value). A total of 1,607 patients (2,202 eyes) with 20 factors were included, and the duration ranged from 0.25 to 24 months. The literature quality was evaluated by the NOS, which showed 7’ in 3 articles [23, 27, 28], 8’ in 8 articles [17, 18, 20–22, 24–26], and 9’ in the remaining ones [9, 19]. All were larger than 6 points, indicating the good quality of the included studies.

**Table 1:** Characteristics and quality of studies included in this systematic review

Article	Design	Country	Age	Duration (Month)	Size/eyes (L/N/H)	Method	Factor	NOS (S + C + E = T)
Qi GW 2020 [23]	CSS	China	27.2 ± 6.4	3	22/86/10	Pentacam	[(1)(2)(4)(8)] (7)(10)(11)(12)	4 + 1 + 2 = 7
Wang J 2021 [24]	CSS	China	24.40 ± 5.76	12	4/68/8	Pentacam	[(1)(2)(17)] (16)	4 + 2 + 2 = 8
Xiong Y 2020 [27]	CSS	China	28.40 ± 7.40	0.25	10/246/20	AS-OCT	[(1)(2)(3)] (4)(5)(6)(7)(8) (9)(10)(11)(12)	3 + 1 + 3 = 7
Wu Y 2022 [25]	CSS	China	23.60 ± 4.35	3–6	0/39/19	Pentacam	[(1)(2)(5)(10)(9)(13)] (4)(6)(7)(11)(12)(14)(15)	3 + 2 + 3 = 8
Li N 2019 [22]	CSS	China	25.17 ± 4.57	24	41/126/21	Pentacam	[(1)(2)(4)(8)] (7)(10)(11)	4 + 1 + 3 = 8
Zhang X 2021 [28]	1:1 CCT	China	18~47	12–24	76/140/58	AS-OCT	[(1)(2)(3)(6)(8)(10)(16)](7)(11)	4 + 1 + 2 = 7
Xi H 2022 [26]	CSS	China	28.27 ± 7.39	1	12/62/24	AS-OCT	[(1)(2)(3)(4)(5)(6)] (10)	4 + 1 + 3 = 8
Cui TF 2019 [21]	1:1 CCT	China	26.2 ± 8.4	12	38/70/29	Pentacam	[(1)(3)(6)(16)] (2)(7)	4 + 2 + 2 = 8
Kyum KW 2012 [20]	CSS	Korea	26.1 ± 4.0	2	8/93/29	AS-OCT	[(1)(3)] (2)(10)(14)	4 + 2 + 2 = 8
Khan MA 2022 [19]	1:2 CCT	Japan	28.93 ± 5.26	12	27/54/0	AS-OCT	[(1)(17)(18)(12)] (2)(7)(9)(10)(11)(14)(19)	4 + 2 + 3 = 9
Chen Q 2020 [18]	1:2 CCT	China	25.13 ± 5.29	1	0/54/27	Pentacam	[(4)(16)(19)] (1)(2)(3)(6)(7)(8)(10)(14)(17)	4 + 1 + 3 = 8
Cerpa MS 2021 [9]	CSS	Spain	32.27 ± 7.56	4	34/272/54	AS-OCT	[(1)(6)(7)(10)(17)] (2)(9)(11)(19)(20)	4 + 2 + 3 = 9
Alfonso JF 2012 [17]	CS	Spain	31.25 ± 6.91	3	65/227/31	AS-OCT	[**(1)(2)**]	4 + 1 + 3 = 8

CSS, case series study; CCT, case-control study; CS, cohort study. L/N/H, low vault/ Normal vault/ High vault. S + C + E = T, population selectivity + comparability between groups + outcome/exposure factor measure = total score. Influencing factors: (1) ACD; (2) hWTW; (3) LT, lens thickness; (4) ACV, anterior chamber volume; (5) ACA, anterior chamber angle; (6) ICL-size; (7) ICL-SE; (8) AL, axial length; (9) CCT, central corneal thickness; (10) age; (11) Kf (12) Ks (13) LDist, Alpha angle; (14) pupil diameter; (15) Kappa angle; (16) posterior chamber structure, including posterior chamber angle, ciliary processes height (T value), or distance between STS plane and crystalline lens (STSL); (17) crystalline lens, including CLR and LC, its rise and curvature; (18) iris morphology, iris concavity; (19) gender; (20) ATA, angle to angle distance. Factors in ‘[]’ are significant ones in the corresponding literature

### Meta-analysis

**Fig. 2:**
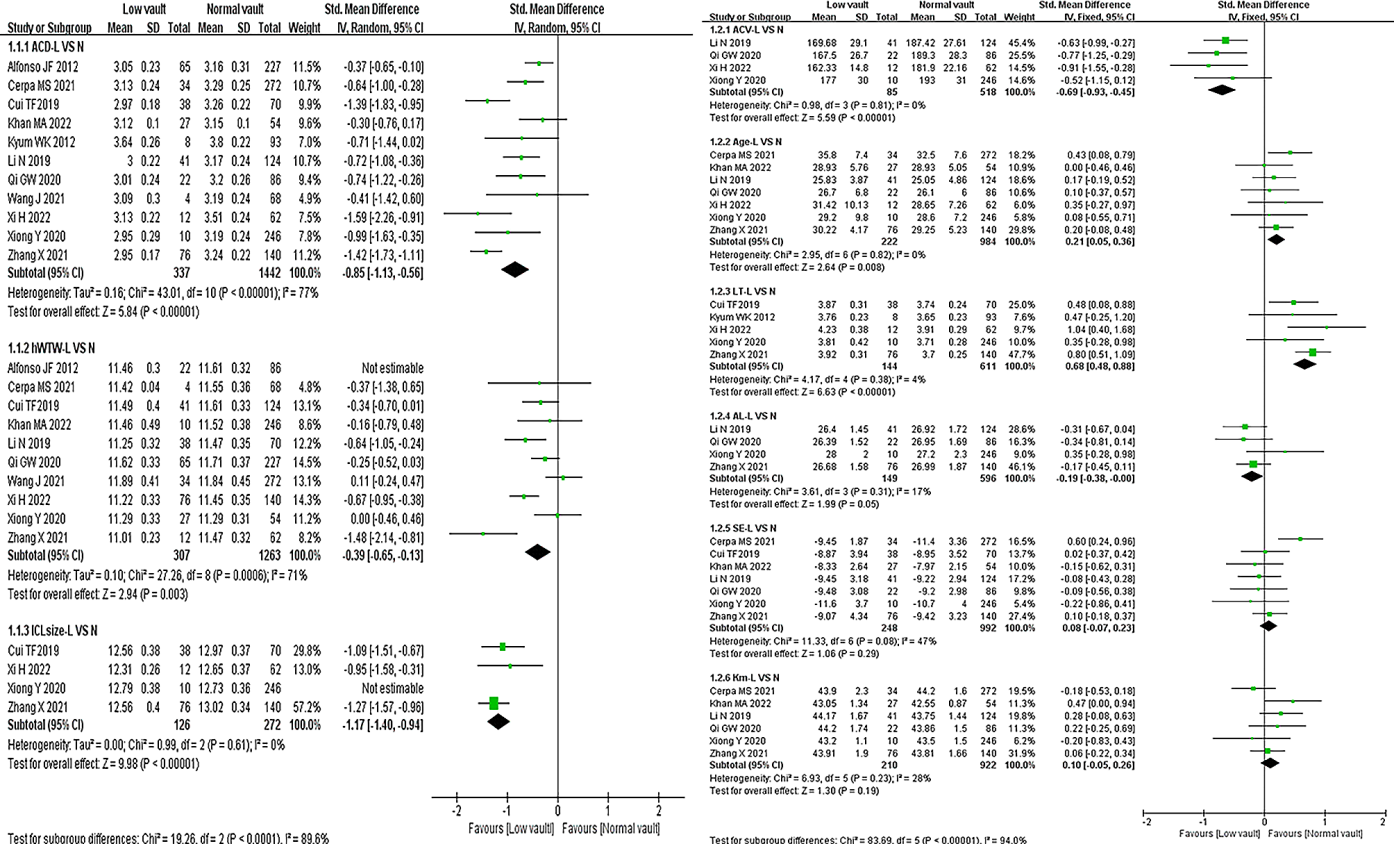
Meta-analysis of the effect of factors on low vault after ICL surgery (L VS N, low vault group vs. normal vault group) Each square indicates a study, and the area of squares is proportional to the weight. The diamond represents the pooled SMD and 95% CI.

Low vault vs. Normal vault (L VS N, Fig. 2): A total of 11 studies [9, 17, 19–24, 26–28] involved ACD in the included articles, with a significant heterogeneity (*P* < 0.05, *I*^*2*^ = 77%), and random-effects model showed that ACD was a protective factor for the postoperative low vault [*SMD*=-0.85, *95% CI* (-1.13, -0.56), *P* < 0.00001]; similarly, hWTW and ICL-size were also protective. Four studies [22, 23, 26, 27] involved anterior chamber volume (ACV) without significant heterogeneity (*P* = 0.81, *I*^*2*^ = 0%), fixed-effects model proved that ACV was a protective factor for low vault [*SMD*=-0.69, *95% CI* (-0.93, -0.45), *P* < 0.00001]; idem, the effect values (SMD) and 95% CI of age and lens thickness (LT) were > 0, which were risk factors. In contrast, the total effect values of the forest plots of axial length (AL), ICL-spherical equivalent (ICL-SE), and Km all crossed the null line and had no significant effect.

**Fig. 3:**
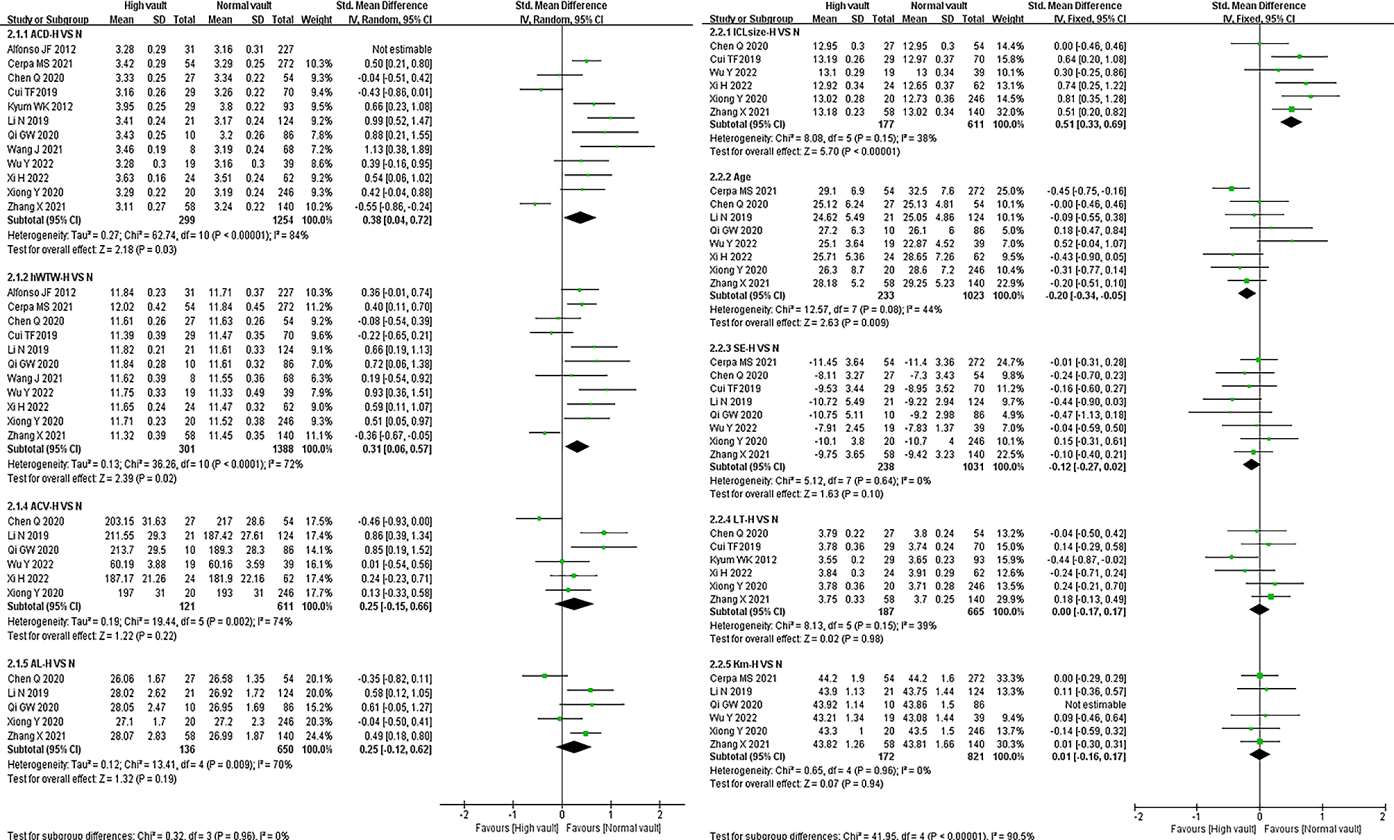
Meta-analysis of the effect of factors on high vault after ICL surgery (HVS N, high vault group vs. normal vault group)

High vault vs. Normal vault (H VS N, Fig. 3): A total of 12 studies [9, 17, 18, 20–28] involved ACD which was a risk factor for the postoperative high vault [*SMD* = 0.38, *95% CI* (0.07, 0.68), *P* = 0.02], with a significant heterogeneity (*P* < 0.00001, *I*^*2*^ = 83%). In the same way, hWTW was also a risk factor, with ACV and AL having no obvious effect. ICL-size referred in Six studies [18, 21, 25–28] with insignificant heterogeneity (*P* = 0.15, *I*^*2*^ = 38%), which was a risk factor for high vault [*SMD* = 0.51, *95% CI* (0.33, 0.69), *P* < 0.00001]; idem, the SMD and 95% CI of age were < 0, which was a protective factor, while ICL-SE, LT, and Km all had no obvious effect.

### Sensitivity analysis, meta-regression and subgroup analysis

Table 2 mainly lists studies with significant changes after a one-by-one exclusion and a significant reduction in heterogeneity (*I*^*2*^ = 0%, *P* = 0.61) when the ICL-size was analyzed at low vault, excluding the study of Xiong et al. [27]. Results after switching to a fixed-effects model showed [*SMD*=-0.42, *95% CI* (-0.50, -0.35), *P* < 0.00001], indicating that it was the main source of heterogeneity, but there was no significant difference in the meta-analysis, and the combined results were stable. After the remaining studies were excluded one by one, the heterogeneity did not change obviously; all the meta-analyses had *P* < 0.10, indicating that the stabilities of their combined results were still in an acceptable range.

**Table 2:** Sensitivity analysis

Factor	Article	Heterogeneity test			Meta-analysis	
		*I*^2^(%)	*P*		SMD (95% CI)	*P*
**Low vault vs. Normal vault**						
ICL-size	Primary Meta	81	0.001		-0.83 (-1.38, -0.28)	0.003
	(Removed) Xiong 2020^27^	**0**	0.61		-1.17 (-1.40, -0.94)	< 0.00001
	(Removed) Zhang 2021^28^	82	0.004		-0.64 (-1.40, 0.11)	0.09
	(Removed) Cui 2019^21^	88	0.0003		-0.71 (-1.56, 0.13)	0.10
Age	Primary Meta	0	0.82		0.21 (0.05, 0.36)	0.008
	(Removed) Cerpa MS 2021	0	0.96		0.15 (-0.01, 0.32)	0.07
**High vault vs. Normal vault**						
hWTW	Primary Meta	72	< 0.0001		0.31 (0.06, 0.57)	0.02
	(Removed) Wu 2022^25^	71	0.0004		0.26 (0.00,0.51)	0.05

Meta-regression analysis was performed to screen for factors that might influence heterogeneity: country and design were factors for heterogeneity of ACD and ICL-size respectively on low vault. (*Z* = 2.64, -1.85, *P* = 0.008, 0.064). Meanwhile, design on high vault was a factor for ACD heterogeneity (*Z*=-4.00, *P* < 0.001). The factors with regression *p* < 0.10 were selected for further subgroup analysis to explore the significant factors of heterogeneity and the differences between subgroups. (Table 3).

**Table 3:** Meta-regression

Meta_es	ACD: L VS N			ICLsize: L VS N			ACD: H VS N		
	Conf	Z	*P*	Conf	Z	*P*	Conf	Z	*P*
Year	-0.042	-1.05	0.292	-0.092	-0.30	0.768	-0.016	-0.33	0.743
Nation	0.218	2.64	0.008^*^	—	—	—	0.047	0.34	0.735
Design	0.091	0.41	0.685	-0.794	-1.85	0.064^*^	-0.820	-4.00	0.000^*^
Method	0.029	0.09	0.928	0.378	0.45	0.653	-0.132	-0.41	0.685
Time	-0.125	-0.74	0.462	-0.450	-1.60	0.110	-0.155	-0.82	0.412
NOS	0.293	1.58	0.115	-0.420	-0.56	0.573	0.190	0.70	0.483
Myopia	-0.506	-1.61	0.107	—	—	—	-0.572	-1.60	0.109
ICL-V	0.430	1.24	0.216	—	—	—	0.173	0.41	0.682

*, represents that the corresponding factor is statistically significant (test level: α = 0.10);

—, means that the corresponding factor does not differ between groups or there is covariance

**Fig. 4:**
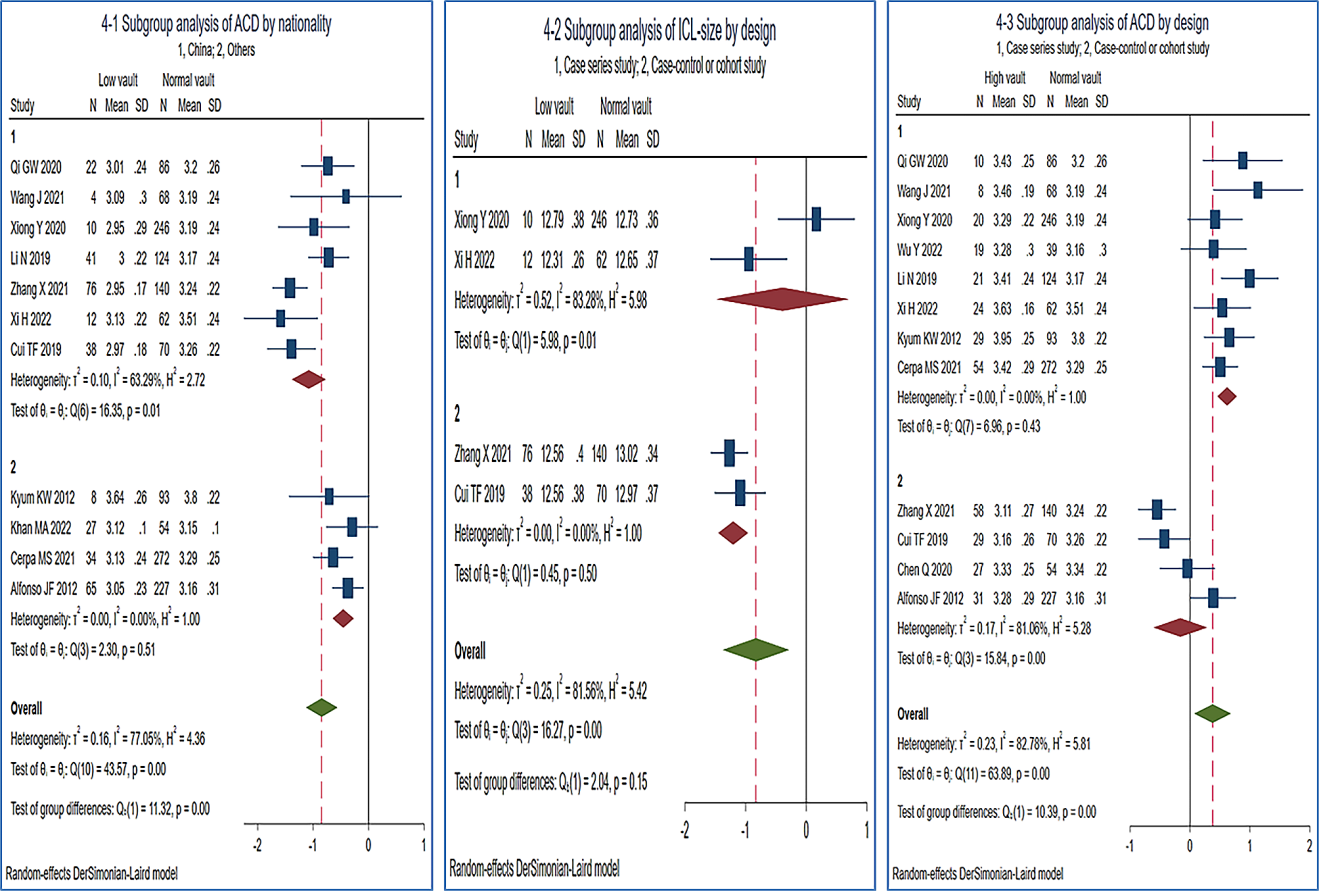
Subgroup analysis

Subgroup analysis: Country was not a main source of ACD heterogeneity on low vault (*I* [2]_*Chinese, others*_=63.29%, 0%, *P* = 0.01, 0.51), and as shown in Figs. 4-1, the small diamond-shaped squares all fell to the left of the invalid line and do not intersect, which means ACD was a protective factor for low vault both in Chinese and others (Japanese, Korean, and Hispanic). Similarly, design was neither a main source of ICL-size heterogeneity on low vault nor of ACD heterogeneity on high vault. Meanwhile, the protective effect of ICL-size on low vault was primarily due to the findings of case-control or cohort designs, but the risk of ACD on high vault was mainly derived from case series studies (Figs. 4-2, 4-3).

### Descriptive analysis

Other factors were not meta-analyzed because of too few studies (< 3), covering 4 cohort studies [18, 19, 21, 28] and 3 case series ones [9, 24, 26], involving a total of 5 factors including anterior chamber angle (ACA), hSTS, T value, crystalline lens rise (CLR) and gender. The study of Xi et al. [26] showed a positive association between ACA and vault at one month postoperatively (*r* = 0.412, *P* < 0.001), and its weight was second only to WTW and LT. Chen and Cerpa [9, 18], on the other hand, denied it (both *P* > 0.05). Cui et al. [21] emphasized the necessity of posterior chamber structure for ICL size selection; Chen et al. [18] held against it (*P* = 0.09). They [18, 21] both supported the contribution of T value to vault, but Cui believed that it contributed the most to vault. Significant differences in CLR have been demonstrated [9, 19, 24] (all *P* < 0.001), and Wang et al. [24] found a negative correlation between CLR and vault (*r*=-0.509, *P* < 0.01). Included studies [9, 18, 19] showed no difference between genders except for Chen (*P* = 0.04).

### Publication bias

**Fig. 5:**
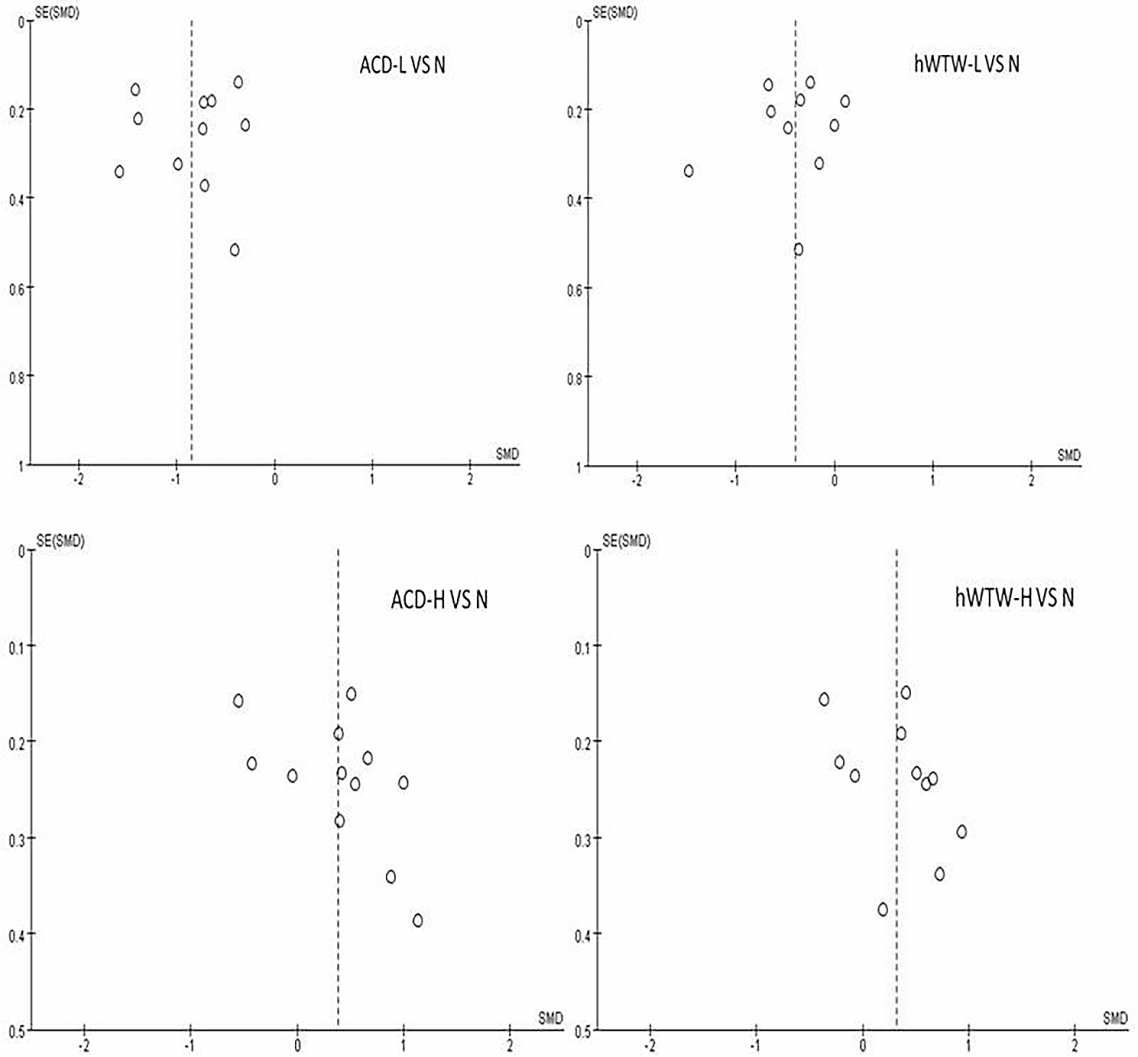
Funnel diagram

Publication bias was assessed by Egger test for individual factors that was included in more than 10 articles. The results suggest that there was no publication bias (*Z*=-0.04, -0.70, 1.94, 1.34; *P* = 0.971, 0.484, 0.052, 0.182) for ACD and hWTW in either the low or high vaults groups (Fig. 5).

## Discussion

The reliability of the results needs to be verified [29]. First, the study of Xiong et al. [27] was a significant factor in analyzing ICL-size heterogeneity at low vault. Next, subgroup analyses showed that none of the factors was the main source of respective heterogeneity. The protective effect of ACD on low vault applied to different countries. There was no difference in the effect of ICL-size on low vault among design subgroups, and its protective role was derived mainly from case-control or cohort studies. The effect of ACD on high vault was relatively heterogeneous on case-control and cohort studies, and its risk role was mainly derived from case series ones. There was no publication bias.

### Possibility analysis of results generation

Age becomes a dual factor, suggesting the need to focus on overall factors [30]. The eye undergoes age-related changes, such as CLR enlargement, pupil narrowing, and iris deformation [31, 32], which have an impact on vault. Studies [17, 33] have found a decrease in vault of approximately 5 μm per year, making age a protective factor for high vault. ACV narrowing or LT thickening can cause low vault in one direction, implying that both are more common (e.g., glaucoma, cataract, and diabetes) [34, 35], and have a greater impact on posterior chamber structure [36]. Posterior chamber structure, one of the most important factors affecting vault [21], reverse compression of the iris concave surface can result in a reduced vault (< 100 μm) [19]; a small posterior chamber angle can restrict ICL contact with the deep surface of the ciliary sulcus and result in a high vault. Thus, scholars proposed to use the direct parameters, LC and hSTS, which reflect the size of the ciliary sulcus space, as novel indicators to select ICL [37]. Also, the significant effects of ACA, hSTS, T value and CLR on vault corroborated the importance of posterior chamber structure on vault, which needs to be focused on its accurate measurement. None of the changes in AL [38], Km or ICL-SE have a significant effect on vault, suggesting a limited influence of non-posterior chamber structure. The short duration (only 1 week) of the study of Xiong et al. [27] may explain why it is the main source of heterogeneity. The difference in results between meta-regression and subgroup analysis is related to the different principles of the two methods [39]. The design belongs to methodological heterogeneity and the country is classified as clinical one [40].

### Applications and implications

Only by making primary and secondary references to the factors, it is expected to improve the accuracy. Later studies could include multi-center data, and the factors with greater weight can be incorporated into regression mathematical models to quantitatively predict and guide clinical practice.

The results of individual factors were strongly influenced by the number of articles; the study sources may have regional bias; and a few main sources of heterogeneity were not effectively identified despite meta-regression and subgroup analysis. However, this study is close to clinical controversy, with comprehensive content, rigorous screening and a large number of cases, and selected initial data of the included literature, while in-depth exploration of the main sources of heterogeneity, and the results were stable and reliable.

## Conclusions

Vault after ICL is related to multiple factors, especially anterior segmental biologic parameters, and our findings emphasize the similarities, differences and weights of influencing factors. ACD, hWTW, ICL-size, and age are the dual factors of vault; ACV and LT are the unidirectional factors; while AL, ICL-SE, and Km have little influence. Except for gender, all other factors tended to be significant. It could provide preliminary guidance for the consideration of factors in the size selection of ICL or intraoperative adjustment, which is helpful to improve the safety and visual quality of ICL.

## Data Availability

Data used in the analyses can be found in the published article, which were listed in the references of this manuscript.

## Abbreviations

ACA: Anterior chamber angle
ACD: Anterior chamber depth
ACV: Anterior chamber volume
AL: Axial length
CLR: Crystalline lens rise
hSTS: Horizontal sulcus to sulcus
hWTW: Horizontal corneal white-to-white
ICL: Implantable Collamer Lens
LT: Lens thickness
PRISAM: Preferred reporting items for systematic reviews and meta-analyses
SE: Spherical equivalent
